# Comparison of microbial colonization rates between central venous catheters and peripherally inserted central catheters

**DOI:** 10.1186/s13756-023-01285-1

**Published:** 2023-08-07

**Authors:** Vassiliki Pitiriga, John Bakalis, Kalliopi Theodoridou, Evangelia Dimitroulia, George Saroglou, Athanasios Tsakris

**Affiliations:** 1 Department of Microbiology, Medical School, National and Kapodistrian University of Athens, 75 Mikras Asias Street, 11527 Athens, Greece; 2 Department of Internal Medicine, Metropolitan Hospital, 9 Ethnarchou Makariou Street, 18547 Athens, Greece

**Keywords:** Catheterization, Central venous catheter, Colonization, Bloodstream infection, Central line-associated bloodstream infection, Peripherally inserted central catheter

## Abstract

**Background:**

Central venous catheters (CVCs) and peripherally inserted central catheters (PICCs), have been widely used as intravascular devices in critically ill patients. However, they might evoke complications, such as catheter colonization that has been considered as predisposing factor for central line-associated bloodstream infections (CLABSIs). Although numerous studies have compared the risk of bloodstream infections between PICCs and CVCs, comparative studies on their colonization rates are limited.

**Objectives:**

The episodes of catheter colonization in critically ill patients with CVCs or PICCs were retrospectively analysed during a two-year period in a Greek tertiary care hospital and colonization rates, microbial profiles and antimicrobial susceptibility patterns were compared.

**Methods:**

Clinical and laboratory data of consecutive hospitalized critically-ill patients who underwent PICC and CVC placement between May 2017–May 2019 were analysed. All catheters were examined by the semiquantitative culture technique for bacterial pathogens, either as a routine process after catheter removal or after suspicion of infection. Species identification and antimicrobial resistance patterns were determined by the Vitek2 automated system.

**Results:**

During the survey period a total of 122/1187 (10.28%) catheter colonization cases were identified among CVCs and 19/639 (2.97%) cases among PICCs (*p* = 0.001). The colonization rate was 12.48/1000 catheter-days for the CVC group and 1.71/1000 catheter-days for the PICC group (*p* < 0.001). The colonization rate per 1000 catheter-days due to multidrug-resistant organisms (MDROs) was 3.85 in all study cases, 7.26 (71/122) in the CVC group and 0.63 (7/19) in the PICC group (*p* < 0.001). Within the CVC group, the most common microorganism isolated was MDR *Acinetobacter baumannii* (*n* = 38, 31.1%) followed by MDR *Klebsiella pneumoniae* (*n* = 20, 16.4%). In the PICC group, the predominant microorganism isolated was *Candida* spp. (*n* = 5, 23.8%) followed by MDR *K. pneumoniae* and MDR *A. baumannii* in equal numbers (*n* = 3, 14.2%).

**Conclusion:**

PICC lines were associated with significantly lower colonization rates comparing to the CVC ones. In addition, patterns of microbial colonization revealed a trend over the predominance of MDR gram-negatives in CVCs suggesting that PICCs might be a safer alternative for prolonged inpatient intravascular access. Prevention programs directed by local microbial ecology may diminish catheter colonization rates and CLABSIs.

## Introduction

Central venous catheters (CVCs) as medical devices are ubiquitous in healthcare setting due to their proficient function in intravenous drug administration and hemodynamic monitoring [1, 2]. Peripherally inserted central catheter (PICC), an alternative option for intravascular access, play also an important role in the management of hospitalized patients, especially in intensive care unit patients [3]. PICCs exerts various advantages compared with the traditional CVCs, such as effective placement with no any organ damage, low cost, and capability for long-term vein access [4]. Regarding central line-associated bloodstream infections (CLABSIs) and catheter-related bloodstream infections (CRBSIs), it is generally considered that PICCs display lower risk of bloodstream infection, compared to CVCs [5, 6]. Infectious complications arise upon the colonization of catheter tips by microorganisms progressing along both the outer surface and the inner lumen of the catheter, mainly originated from the skin flora, but also from catheter care by medical stuff [7]. Therefore, catheter colonization is considered of great importance since it serves as a harbinger of CLABSIs and may be used as an indication for timely detection of a population at-risk, as suggested by previous studies [8, 9].

The majority of the studies over the last decades have emphasized on incidence rates of catheter-related blood infections instead of catheter colonizations, [10] since bacteraemias frequently increase patients’ morbidity, prolong hospitalization, and augment medical costs. Only limited data exist about the comprehensive rate of catheter bacterial colonization; they refer only to CVCs and not PICCs [11–13] as well as for specific populations [14]. Moreover, studies referring to CVC colonization rates report data only for certain pathogens of major clinical importance (e.g. fungi, staphylococci.) [15, 16]. Furthermore, no data exist regarding differences in microbial patterns among colonized PICCs and CVCs.

The current retrospective study was performed in order to assess catheter colonization rates of both CVCs and PICCs in critically ill patients, based on routine tips cultures after catheter withdrawal, aiming not only to provide the full colonization microbial profiles of both types of catheters but also to compare the microbial distribution along with the MDROs rates.

## Materials and methods

### Study design

We performed a retrospective analysis of data collected from the medical records and microbiology laboratory findings of consecutive adult critically-ill hospitalized patients who underwent PICC and CVC placement. The survey was undertaken between May 2017 and May 2019 in Metropolitan General Hospital, a large tertiary care hospital of Piraeus, Attica Prefecture, Greece. This observational study was approved by the institutional review board.

### Data collection

After insertion, catheters were checked using a check-box form containing the patient’s diagnosis, operator’s name, site chosen, date placed and removed, date of intensive care units (ICU) discharge or death, mechanical ventilation, arterial catheters, parenteral nutrition, and daily clinical assessment (e.g., discharge, erythema, and tenderness) of possible catheter infection. The operator inserting the catheter entered the initial data; nurse personnel entered data the following days while the infection control nurse monitored data collection 3–4 times per week. Data was retrospectively collected from two different data sources: (1) medical database (for demographic and clinical data related to the patient’s admission and clinical course) and (2) Clinical laboratory and hospital infection control team database (for blood culture and antibiotic susceptibility results). The participants of the study were patients that had routinely removed catheters and also had (a) no signs of local inflammation (redness, swelling, and pain with pressure or tapping on the insertion site) and (b) no clinical symptoms of bacteremia. We also included patients with suspicion of bacteremia that was not laboratory confirmed (negative blood cultures).

### CVC and PICC insertion protocol

In our hospital triple lumen, non-antibiotic impregnated catheters (Arrow model, total provided by Arrow®/Teleflex®, Wayne, USA) are mainly used. Double lumen catheters (Arrow®/Teleflex®, Wayne, USA), are also used but in a lower percentage, particularly in patients that do not require complex therapeutic interventions. The choice of the site of insertion was left to the discretion of the physician caring for the patient. Maximal sterile barrier precautions (large sterile drape; surgical hand antisepsis; and mask, cap, sterile gloves, and gown) were used at catheter insertion according to CDC recommendations [17].

### Catheter care protocol

Standardized CVC/PICC care practices were implemented by a highly proficient nursing staff. Every couple of days or earlier if clinically required, the nursing staff changed the dressing, cleaned the skin site and the catheter hub with iodine solution, and changed the intravenous accessory tubing. Catheters were removed when (a) there was evidence or suspicion of infection, (b) when the catheter was no longer required.

### Culture techniques

All catheters were examined for the presence of pathogens either as a routine after removal or after suspicion of infection. It is our institution’s policy to routinely test by culture all catheter tips after catheter removal. This procedure has been approved by the Hospital Scientific Board, in order to predict and promptly take actions in cases of potential occurrence of bloodstream infections following the removal of the catheters.

In cases where no clinical symptoms of bacteremia were presented, no blood cultures were ordered along with the tip cultures. In cases of potential CLABSI or CRBSI, blood cultures were accompanied tip cultures. After disinfecting skin around the catheter entry site, the proximal 4–5 cm part of the tip was cut off using sterile scissors. The specimen was placed in a sterile container and transported to the department of microbiology within 15 min at room temperature. The intradermal and intravascular portion of the catheter was analyzed by the semiquantitative culture technique described by Maki et al. [18] According to Maki’s technique, catheter-tip culture is considered positive in the presence of ≥ 15 colony-forming units (CFU) growth of any organism. Blood cultures were incubated in Becton Dickinson Bactec (BD Bio-sciences, USA) in aerobic and anaerobic broth media. Identification of isolates and antimicrobial resistance patterns were determined by the VITEK®2Automated Compact System (BioMérieux Co., France). E-test (BioMérieux Co.) was performed as an additional test, in order to confirm the resistance phenotypes reported by the VITEK System, according to the standard laboratory procedures.

### Definitions

*CVC* was defined as any central venous access device inserted into the internal jugular, subclavian, or femoral vein that terminated in the inferior vena cava or right atrium.

*PICCs* were defined as catheters inserted in the basilic, cephalic, or brachial veins of the upper extremities with tips that terminated in the superior vena cava or right atrium.

*Catheter-days* was defined as the number of CVCs/PICCs presents among all units’ patients at 08:00 h each morning.

*Multidrug-resistant organisms (MDROs)* were defined as **s**pecies of microorganisms that exhibit antimicrobial resistance to at least one antimicrobial drug in three or more antimicrobial categories. [19] This definition concerns both gram-positive and gram-negative bacteria.

*Catheter colonization* was considered the presence by a semi-quantitative culture of ≥ 15 CFU of at least a single organism per catheter, according to Maki et al. [18]

### Statistical analysis

Descriptive analysis to characterize patients’ population were reported as count (percent) or mean value (+ / − standard deviation) for qualitative and quantitative variables, respectively, and were compared between the two groups using Chi-square test or Student’s t-test, as appropriate. A two-sided *P* value of ≤ 0.05 was considered as statistically significant.

## Results

### Patients’ characteristics

A total of 1187 CVCs were placed for 9774 catheter/days and 639 PICCs for 11,110 catheter-days were inserted during the two-years period. The total patients’ demographic characteristics are presented in Table 1. No significant differences were determined among the two patient groups. The majority of the study population was catheterized with three-lumen catheters. Two-lumen catheters were placed in only three cases. The mean duration of indwelling time was 20.47 ± 10.1 days (range: 3–87 days) in PICCs and 14.4 ± 8.5 days (range: 2–40 days) in CVCs. The etiology for catheter removal was end of use (85.5%), suspicion of infection (10.7%), and other (3.8%). In cases with suspicion of infection (potential CLABSI or CRBSI), all blood cultures accompanied by tip cultures gave negative results.

**Table 1:** Demographical characteristics and indicators of illness severity among CVC/PICC groups

Characteristics	CVC patients (*n* = 122)	PICC patients (*n* = 19)
	N (%)	N (%)
Demographical		
Age, mean ± SD, (years)	58.02 ± 17.4	62.28 ± 14.2
Gender (M/F)	81/41	11/8
Obesity	48 (39.3)	6 (31.5)
Indicators of illness severity		
ICU admission	58 (47.5)	8 (42.1)
APACHE score	14.8 ± 8.2	13.4 ± 7.5
Mechanical ventilation	78 (63.9)	8 (42.1)
In-hospital mortality	10 (8.2)	2 (10.5)
Sepsis	8 (6.5)	2 (10.5)
Duration of catheter use (mean ± SD)	14.4 ± 8.5	20.47 ± 10.1
Length of hospital stay before IV catheter (mean ± SD)	50.2 ± 21.4	39.4 ± 15.4

IV, intravenous; SD, standard deviation; M/F, Male/Female

### Colonization rates of CVCs and PICCs

A total of 122 (10.28%) catheter colonization cases were identified among CVCs, and 19 (2.97%) cases among PICCs during this period (*X*^2^, *p* = 0.001). The colonization incidence rate was 12.48 per 1000 catheter-days for CVC group and 1.71 per 1000 catheter-days for PICC group (T-test, *p* < 0.001). The colonization rate per 1000 catheter-days due to multidrug-resistant organisms (MDROs) was 3.85 in total study cases, 7.26 (71/122) in CVC group and 0.63 (7/19) in PICC group (T-test, *p* < 0.001; Table 2).

**Table 2:** Colonization incidence rate among CVC/PICC groups

	PICC	CVC	*P* value
No of catheters	639	1187	
No of catheter-days	11110	9774	
MDR pathogens, No (%)	7 (1.1)	71 (6.0)	X^2^ = 22.6 *p* < 0.001
MDR pathogens rate (per 1000 catheter-days)	0.63	7.26	T-test *p* < 0.001
non-MDR pathogens, No (%)	12 (1.9)	51 (4.3)	X^2^ = 6.8 *p* < 0.008
non-MDR pathogens rate (per 1000 catheter-days)	1.08	5.22	T-test *p* < 0.001

No, number; MDR, multidrug resistant

### Microbial distribution patterns

From all positive catheter tip cultures (*n* = 141 patients), twenty different species of microorganisms were recovered; gram-negative bacteria (*n* = 98, 69.4%), gram-positive bacteria (*n* = 25, 17.4%) and fungi (*n* = 19, 13.2%). The five most common microorganisms were *Acinetobacter baumannii* (*n* = 41, 28.6%), *Klebsiella pneumoniae* (*n* = 25, 17.4%), *Candida* sp. (*n* = 19, 13.2%) *Pseudomonas aeruginosa* (*n* = 14, 9.8%) and *E. coli* (*n* = 9, 6.3%). Two (1.4%) of PICC catheter tip cultures were polymicrobial.

**Fig. 1:**
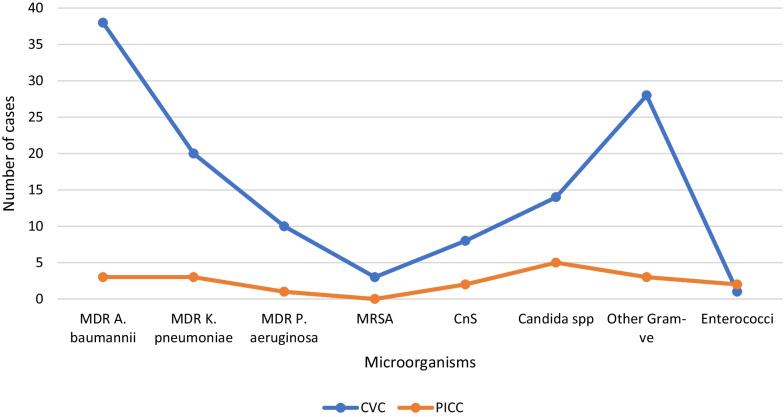
Microbial distribution among CVC/PICC groups. CnS, Coagulase negative staphylococci; MRSA, methicillin-resistant *Staphylococcus aureus*; Gram-ve, Gram-negative; MDR, multidrug-resistant

The microbial distribution in CVC and PICC groups are presented in Table 3. The microorganisms isolated from colonized CVCs were gram-negative bacteria (*n* = 90, 73.7%) gram-positive bacteria (*n* = 18, 14.7%), and fungi (*n* = 14, 11.6%). The microorganisms isolated from colonized PICCs were gram-negative bacteria (*n* = 8, 38%), gram-positive bacteria (*n* = 8, 38%) and fungi (*n* = 5, 24%). Within CVC group, the most common microorganism isolated was MDR *A. baumannii* (*n* = 38, 31.1%) followed by MDR *K. pneumoniae* (*n* = 20, 16.4%). In PICC group, the predominant microorganism isolated was *Candida spp.* (*n* = 5, 23.8%) followed by MDR *K. pneumoniae* and MDR *A. baumannii* in equal numbers (*n* = 3, 14.2%) (Fig. 1).

**Table 3:** Microbial distribution among CVC and PICC colonized groups

Isolates	CVC No of isolates (%)*	PICC No of isolates (%)*
Gram positive bacteria		
*S. aureus*	1 (0.8)	–
*S. haemolyticus*	3 (2.4)	1 (4.7)
*S. mitis*	1 (0.8)	–
*S. salivarius*	–	1 (4.7)
*E. faecium*	–	2 (9.5)
Other coagulase-negative staphylococci	8 (6.5)	3 (14.2)
MRSA	3 (2.4)	1 (4.7)
*Bacillus* spp*.*	1 (0.8)	–
Gram-negative bacteria		
MDR *A. baumannii*	38 (31.1)	3 (14.2)
MDR* K. pneumoniae*	20 (16.4)	3 (14.2)
MDR *P. aeruginosa*	10 (8.2)	–
*E. coli*	9 (7.3)	–
*S. marcescens*	3 (2.4)	–
*E. cloacae*	2 (1.6)	–
*K. pneumoniae*	2 (1.6)	–
*M. morganii*	1 (0.8)	–
*P. aeruginosa*	3 (2.4)	1 (4.7)
*P. mirabilis*	2 (1.6)	1 (4.7)
Fungi		
*Candida* spp.	14 (11.4)	5 (23.8)
Total	122	21

*The total number was not 141 owing to polymicrobial infections

## Discussion

This is the first study providing information about PICCs colonization bacterial profile, to assess the incidence rates of all bacteria isolated by culture from CVCs, and also to compare the full microbial profile along with MDROs rates between colonized PICCs and CVCs. In clinical studies designed on the prevention of catheter-related infections, catheter-tip colonization is considered as factor of crucial importance since it has been frequently used as a surrogate end-point for occurrence of CLABSIs [20, 21]. This decision has been based on observations that, in patients who have an indwelling catheter in place and develop bloodstream infection, the catheter is more possible to be the cause of bacteraemia, provided that the culture of the catheter tip yields the same microbe as blood culture. Colonization of intravascular catheters can occur via the skin of the patient, the hospital environment or contaminated fluids [22]. Catheters placed for no longer than 8 days are frequently colonized by skin microorganisms, followed by microbes from the hub/lumen. In cases of long-term catheters (> 8 days), hub is the most common source of colonization, followed by the skin flora [23]. With the emergence of multidrug-resistant pathogens in CLABSIs and CRBSIs, the treatment of catheter-related infections and the selection of appropriate antibiotic treatment has become more difficult [24–26]. Taking into consideration that MDROs are accountable for 20–67% of all CLABSIs [27], it seems crucial to identify the optimal management strategies for catheterized patients. In this context, characteristics and distribution of microorganisms in colonized catheters need to be accurately determined and timely updated as to direct to optimal clinical practices.

Regarding CVCs, the findings of our study indicated that *Acinetobacter baumannii* predominated among isolated microorganisms. Most recent studies report gram-positive bacteria, predominantly *Staphylococcus* and *Streptococcus,* to account for most CVC colonization episodes, followed by gram-negative bacteria and *Candida* [28, 29]*.* With the extended application of CVCs the recent decades, the proportion rates of microorganisms present diversity worldwide that could be attributed to differences in geographical regions epidemiology and hospital environments [30]. In our study, the different CVC microbial profile could be attributed to the incidence rates of our nosocomial pathogens, where MDR *A. baumannii* is frequently isolatedfrom critically-ill patients.

In contrast, the microbial distribution of PICCs displayed a different profile, with *Candida* spp. being the microorganism mainly isolated. This could be attributed to the longer duration of catheter placement in patients with PICCs compared with those with CVCs [31]. Apart from the long-term use of catheters, other important risk factors for candidemia are often total parenteral nutrition, frequent use of broad-spectrum antibiotics, toxic chemotherapeutic agents, complex surgical procedures and corticosteroids [32–34]. *Candida* species have been reported as one of the most common opportunistic pathogens, while it is reported to be the fourth microbial agent of nosocomial bloodstream infection among immunocompromised patients who were hospitalized in the United States the last two decades [35].

In our study, we did not apply advanced molecular techniques such as high-throughput sequencing. Instead, all reported microorganisms were isolated by culture-dependent methods. Traditional methods are known to support microorganisms that grow quickly under standard laboratory conditions in culture media. In addition, the sensitivity of the Maki semi-quantitative method may also be reduced since some bacterial species may compete with others for nutrients or they may even inhibit the growth of other microbes. Therefore, our colonization rate of 10.28% is significantly lower than recent studies applying molecular methods [36]. Indeed, culture-independent molecular approaches can recognise the composition of complex microbial communities, and are nowadays being applied to detect “novel” pathogens and to depict the polymicrobial nature of indwelling catheters colonization and CLABSIs [37]. However, in our opinion, the detection of low-abundance species by these techniques is going to complex the evaluation of their role in terms of clinical importance.

Specific limitations should be acknowledged in the present study. The retrospective data analysis of the two patient populations probably contains potential selection bias in terms of patient characteristics, severity of illness, patients’ treatments among the two groups. However, when examining the demographical characteristics of both groups (presented in Table 1), no significant differences exist in underlying diseases or medical history. This is reasonable, since both PICCs and CVCs are used in our hospital only for severe cases, to ensure safety and appropriate patient management during hospitalization, such as the need for a large and constant replenishment of fluid volumes in hemodynamically unstable patient, in septic patients, in patients that need multiple treatments simultaneously, in cases of heavy surgeries and in multi-trauma patients. Moreover, the difference in APACHE score, which we consider as the most straightforward single variable to use for severity of illness, was not significantly different between the two groups.

## Conclusions

Our results suggest the beneficial use of PICCs compared to CVCs in critically ill patients, in terms of colonization incidence rates, despite their longer indwelling time. Moreover, a significant swift in the epidemiological profile of pathogens towards a high percentage of gram-negative pathogens and specifically MDROs was observed in colonized CVCs.

More studies are needed to explore the relationships between the presence of microorganisms in colonized PICCs and CVCs and the potential risk of future bloodstream infection, possibly through the comparison of the bacterial community parameters between asymptomatic patients with colonized catheters and patients with CLABSIs. Moreover, this knowledge may be valuable in predicting the group of patients that are at risk of developing bloodstream infections and permit triage of them in order to implement specific preventive measures.

## Data Availability

All data generated or analyzed during this study are included in this published article.

## Abbreviations

CLABSI: Central-line associated bloodstream infection
CVC: Central venous catheter
ICU: Intensive care unit
MDRO: Multidrug-resistant organism
PICC: Peripherally inserted central venous catheter
